# Mechanisms and Implications of Age-Related Changes in the Liver

**DOI:** 10.1155/2011/150364

**Published:** 2012-01-24

**Authors:** Victoria C. Cogger, Sarah N. Hilmer, Dmitri Svistounov

**Affiliations:** ^1^Centre for Education and Research on Ageing and ANZAC Medical Research Institute, Concord Hospital and The University of Sydney, NSW 2006, Australia; ^2^Kolling Institute of Medical Research and Departments of Clinical Pharmacology and Aged Care, Royal North Shore Hospital and Sydney Medical School, The University of Sydney, NSW 2006, Australia

As the world's population ages in unprecedented numbers and proportions, management of the basic health needs of an older population is a key challenge [1]. Older age is unquestionably associated with increased vulnerability and susceptibility to disease and disability [2]. Ageing is the major independent risk factor for most diseases of the Western world including atherosclerosis, cancer, and arthritis as well as for the prototypical aging diseases such as dementia and osteoporosis [3]. Despite this, our understanding of how old age predisposes us to disease remains rudimentary [4].

A greater understanding of the ageing process will provide insight into the underlying causes of many diseases and open up new avenues for prevention and therapy. Our research into the underlying causes of ageing led us to examine the liver architecture in great detail. The age-related decrease in liver function is substantial and very relevant for systemic exposure to substrates implicated in disease pathogenesis and ageing. In the past, the liver was considered to be relatively unaffected by ageing and age-related diseases. Functional changes, particularly related to impaired drug metabolism (which is reduced by 40–50% in old age), were attributed only to age-related reduction in blood flow and liver mass [5]. However, as such mechanisms are unable to fully explain age-related impairment of hepatic function and the systemic effects of these changes, much greater investigation into other hepatic factors such as liver blood vessel ultrastructure, immune function, and gene and protein expression is warranted.

This special issue brings together many of the current areas of research in the liver and ageing. It details findings on the systemic role of the liver in health, disease, and treatment options in the setting of old age.

The first paper in this issue addresses the very important area of DNA repair in ageing in the liver. M. Lebel et al. have written an eloquent review that thoroughly explores the role of genetic instability in age-related loss of liver function. The authors postulate that this is an area of great promise in understanding and combating diminished liver function with age.

S. J. Mitchell et al. question the evidence base for the current prescribing guidelines for the common analgesic paracetamol in older people in their manuscript, which examines how poorly understood pharmacodynamics and pharmacokinetics are in the older person. Better understanding is required to reduce the risks both of underdosing this important analgesic and of causing accidental hepatotoxicity.

D. L. Schmucker and H. Sanchez investigate the impaired liver regeneration seen in older people and animal models and conclude that the regenerative capacity of older liver is not impaired, rather the rate of regeneration is reduced. This conclusion has very important implications for the contentious issue of the use of donor livers from older people in liver transplantation.

A. Warren et al. present an ultrastructural study of the liver in old age, with particular focus on the hepatic stellate cell, a cell synonymous with fibrotic liver changes in disease. The study shows for the first time that, while there is lipid engorgement of the cells with ageing, there is no activation. Smooth muscle actin expression, the hallmark of the hepatic stellate cell dedifferentiation into a fibroblast, is seen in many chronic liver diseases but not in old age. This finding yet again shows that ageing changes in the liver are distinct from the pathological processes seen in disease states.

X. He et al. address the important issue of cancer therapy in the older person and how liver-related changes drastically affect the efficacy and toxicity of chemotherapy agents. Due to comorbidities and poor functional status, older people have generally been omitted from drug therapy trials in this area. The authors argue that the key to increasing treatment efficacy and decreasing toxicity in this group is tailoring treatment specifically to individuals with their age and comorbidities foremost in their treatment plan.

Finally the paper by L. Gan et al. examines nonalcoholic fatty liver disease (NAFLD) in ageing. NAFLD is the most common liver disease today and most people diagnosed with NAFLD are aged over 60 years. The authors point out that “Advanced age is associated with disease severity and fibrosis progression; a relatively high proportion of individuals with progressive forms of NAFLD develop cirrhosis by the time they are in their 70s or beyond, although more data are required on the exact risks.” The paper also presents some management strategies for older people with NAFLD.

These articles present the state of knowledge of many aspects of the liver in ageing. We would suggest that they put forward a compelling argument that a thorough understanding of how the liver changes with age is important in disease prevention, modulation, and treatment in the setting of the older person.

*Victoria C. Cogger* *Dmitri Svistounov* *Sarah N. Hilmer*
